# A Comparison of Leg Muscle Oxygenation, Cardiorespiratory Responses, and Blood Lactate between Walking and Running at the Same Speed

**DOI:** 10.3390/sports12020048

**Published:** 2024-02-01

**Authors:** Alexandros Stathopoulos, Anatoli Petridou, Nikolaos Kantouris, Vassilis Mougios

**Affiliations:** Laboratory of Evaluation of Human Biological Performance, School of Physical Education and Sport Science at Thessaloniki, Aristotle University of Thessaloniki, 54124 Thessaloniki, Greece; alexsta8410@gmail.com (A.S.); kantourisn@gmail.com (N.K.); mougios@auth.gr (V.M.)

**Keywords:** gastrocnemius medialis, movement pattern, muscle oxygen saturation, oxygen consumption, vastus lateralis

## Abstract

It is not known whether different gait modes, or movement patterns, at the same speed elicit differences in muscle oxygen oxygenation, expressed as muscle oxygen saturation (SmO_2_). Thus, the aim of this study was to compare the oxygenation of two leg muscles (vastus lateralis and gastrocnemius medialis), as well as the heart rate, respiratory gases, and blood lactate between two gait modes (walking and running) of the same speed and duration. Ten men walked and ran for 30 min each at 7 km/h in a random, counterbalanced order. SmO_2_, heart rate, and respiratory gases were monitored continuously. Blood lactate was measured at rest, at the end of each exercise, and after 15 min of recovery. Data were analyzed by two-way (gait mode × time) or three-way (gait mode × muscle × time) ANOVA, as applicable. Heart rate and oxygen consumption were higher when running compared to walking. SmO_2_ was lower during exercise compared to rest and recovery, in gastrocnemius medialis compared to vastus lateralis, and in running compared to walking. Blood lactate increased during exercise but did not differ between gait modes. In conclusion, running caused higher deoxygenation in leg muscles (accompanied by higher whole-body oxygen uptake and heart rate) than walking at the same speed (one that was comfortable for both gait modes), thus pointing to a higher internal load despite equal external load. Thus, preferring running over walking at the same speed causes higher local muscle deoxygenation, which may be beneficial in inducing favorable training adaptations.

## 1. Introduction

Technological advances in recent years have provided valuable services to sports sciences by facilitating the measurement and monitoring of numerous physiological and biochemical parameters during exercise, with a strong impact on sport performance and human health. Included in these parameters is muscle oxygenation, expressed, among other parameters, as muscle oxygen saturation (SmO_2_) and defined as the percentage of total heme molecules (as part of the hemoglobin, myoglobin, and cytochrome structure) in muscle tissue that carry bound oxygen at the time of measurement.

SmO_2_ is being increasingly measured in exercise as a non-invasive index of muscle metabolism thanks to the development of small wearable devices that use near-infrared spectroscopy to distinguish between oxygenated and deoxygenated heme [1]. Specifically, SmO_2_ reflects the balance between oxygen supply and consumption in muscle and can be indirectly used to evaluate the microvascular reactivity and hemodynamics of muscle tissue [2,3].

The number of studies on muscle oxygenation and exercise has grown steadily in recent decades (quadrupling from 1994–2003 to 2004–2013 and doubling from 2004–2013 to 2014–2023), as a search of the Scopus and PubMed databases shows. Most of these studies have examined endurance exercise, and many have used running and walking. Rissanen et al. [4] reported a linear decrease in vastus lateralis (VL) oxygenation from a speed of 5 km/h (walking) to a speed of 15 km/h (running to exhaustion). Hiroyuki et al. [5] found that, compared to rest, the oxygenation of the VL increased during walking at 4 and 6 km/h, whereas that of gastrocnemius lateralis decreased; then, running at 8 to 16 km/h had a lowering effect on SmO_2_ in both muscles. Likewise, Lee et al. [6] showed that, although VL and gastrocnemius lateralis SmO_2_ generally increased during walking at 3.2 to 6.4 km/h, it generally decreased during running at 8 to 14.4 km/h. Generally, these studies show an increase in muscle oxygenation from rest to walking and a decrease with increasing running speed.

Although the aforementioned studies have examined muscle oxygenation in both walking and running, we could find no study testing the two gait modes, or movement patterns, at the same speed so as to examine whether gait mode per se, rather than speed, is responsible for differences in muscle oxygenation. This is at odds with the fact that researchers have studied, compared, and found interesting differences in other parameters, such as biomechanical, motor, cardiorespiratory, and perceptual ones, between walking and running at the same speed (e.g., [7,8,9]). Adding muscle oxygenation, together with local blood supply and metabolic indices, to these parameters would offer valuable insight and provide a more complete picture of how gait mode affects biological responses to exercise.

Therefore, the aim of this study was to compare the muscle oxygenation and blood supply of two leg muscles, that is, VL and gastrocnemius medialis (GM), along with heart rate, respiratory gas exchange, and blood lactate concentration, between walking and running at a comfortable speed for both gait modes, which, according to the aforementioned [5,6] and other studies (e.g., Ref. [10]), is around 7 km/h. Such a comparison can shed light on how gait mode affects muscle metabolism when the parameters of external load remain the same.

## 2. Materials and Methods

### 2.1. Sample Size Calculation, Participants, and Ethics

This study was conducted in accordance with the Declaration of Helsinki and approved by the Research Ethics Committee of the School of Physical Education and Sport Science at Thessaloniki, Aristotle University of Thessaloniki (approval number EC-8/11 February 2020).

Because, as mentioned above, we could find no study comparing muscle oxygenation between walking and running at the same speed, we had no data to perform a power analysis and deduce the necessary sample size for our study. Thus, we intuitively chose to recruit 10 volunteers and, based on their data, performed an interim power analysis using the G*Power software (version 3.1), which would show us if additional recruitment was necessary. We found a partial η^2^ of 0.374 for the main effect of gait mode on SmO_2_ (see below under Results, Muscle oxygenation). For an α of 0.05, power of 0.8, and average correlation coefficient among repeated measures of 0.4, the software yielded a sample size of 7. For a power of 0.95, the software yielded a sample size of 9. Thus, the study was adequately powered with 10 participants, and no additional recruitment was warranted.

The inclusion criteria for the study were (i) young age (18 to 35 years), (ii) normal body weight (body mass index 18.5 to 25 kg/m^2^), (iii) thickness of the skinfold at placement points of the muscle oxygenation measurement device below 24 mm (so that the thickness of the subcutaneous fat layer was below 12 mm for valid recordings, according to the manufacturer), and (iv) experience in performing moderate-intensity walking and running on a treadmill while wearing an ergospirometry mask. Exclusion criteria were (i) musculoskeletal injury and (ii) chronic disease that could interfere with the execution of the exercise protocol.

Based on the aforementioned criteria, we recruited 10 young, healthy males, aged 21–26 years, all students at the School of Physical Education and Sport Science, Aristotle University of Thessaloniki, who volunteered to participate after being fully informed and signing a consent form. Although we wanted to also include female participants in the study, we were unable to collect a sample of sufficient size, mainly because the thickness of the subcutaneous fat layer at the VL and/or GM in most female volunteers exceeded the limit of 12 mm for valid recordings.

### 2.2. Study Design

Participants visited the laboratory once, having had a light breakfast up to two hours before. Each participant was initially subjected to anthropometric measurements, followed by two consecutive tests with a 15 min interval in between, one involving walking and one involving running. Ergospirometry, SmO_2_ monitoring, and blood lactate measurements were performed during testing and are described in detail below.

### 2.3. Anthropometric Measurements

Body mass was measured to the nearest 0.1 kg using an electronic balance (Seca, Hamburg, Germany), and stature was measured to the nearest 1 cm by a stadiometer fixed to the balance. Body fat percentage was estimated by measuring four-terminal bioelectrical impedance through a Bodystat 1500 apparatus (Douglas, UK). The skinfold thickness of the VL and GM were measured with Harpenden metal calipers from British Indicators (West Sussex, UK) to ensure the validity of the SmO_2_ readings, as the devices used can measure tissue oxygenation at a depth of up to 12 mm.

### 2.4. Exercise Testing

Exercise testing was performed on a treadmill (H/p/cosmos pulsar 3p 4.0) with an integrated ergospirometer (Jaeger Oxycon Pro, Höchberg, Germany) and consisted of 1 min of rest, 30 min of either walking or running at a speed of 7 km/h horizontally, 15 min of passive recovery, 1 min of rest, 30 min each of running or walking at 7 km/h horizontally, and 15 min of passive recovery. The two gait modes were tested in random (after drawing lots) and counterbalanced order (that is, 5 participants started with walking and 5 with running), and participants executed both without any distress. During testing, oxygen uptake ($\dot{V}O_{2}$), non-protein respiratory exchange ratio (RER), and heart rate (HR), through a built-in Polar heart rate monitor, were measured continuously.

### 2.5. Muscle Oxygenation Monitoring

SmO_2_ was measured continuously during testing in each volunteer’s dominant leg with two Moxy portable devices (Idiag, Fehraltorf, Switzerland). According to the manufacturer, Moxy uses a theoretical calibration model (rather than a physical or physiological one) because it allows for the accommodation of a much wider range of physiologic variables. One was placed on the VL, 14 cm above the center of the patella and 3.5 cm outwards of the imaginary line running along the quadriceps. The other was placed on the GM, about 11 cm below the knee joint and 3 cm inwards of the imaginary line running along the gastrocnemius. Data were collected wirelessly by use of the Idiag Moxy software (version 1.0) every 2 s. The so-called total hemoglobin (tHb) was recorded at the same time. tHb is a dimensionless quantity that reflects changes in muscle blood volume; it is not useful in an absolute sense because it depends on the thickness of the fat layer over the muscle, the relative contribution of myoglobin, and the blood volume relative to muscle volume. Thus, it is not safe to use tHb to compare different sites; however, tHb can provide information about changes in blood supply at a single site. In this sense, tHb was monitored with the purpose of adding mechanistic insight into the changes in SmO_2_.

### 2.6. Blood Lactate Measurements

Lactate was measured in capillary blood from a fingertip with a Lactate Scout 4 portable analyzer (EKF Diagnostics, Magdeburg, Germany). Measurements were performed immediately before the onset of each exercise test, immediately after its end, and at the end of recovery.

### 2.7. Calculations and Statistical Analysis

The values obtained from the continuous monitoring of HR, $\dot{V}O_{2}$, RER, SmO_2_, and tHb were averaged every minute. Energy expenditure during exercise was calculated from average $\dot{V}O_{2}$ and RER, based on Péronnet and Massicotte [11]. The distribution of all dependent variables was examined by the Shapiro–Wilk test and was found not to differ significantly from normal. Data are presented as the mean ± standard deviation. Significant differences in HR, $\dot{V}O_{2}$, RER, tHb in each muscle, and lactate were calculated by two-way (gait mode × time) ANOVA. Significant differences with respect to SmO_2_ were tested by three-way (gait mode × muscle × time) ANOVA. Effect sizes for significant main effects and interactions were determined as partial η^2^ and were classified as small (0.01–0.058), medium (0.059–0.137), or large (>0.137) according to Cohen [12]. Average RER and energy expenditure during walking and running was compared through a paired Student’s *t* test. To examine the possible presence of an order effect, the aforementioned analyses were repeated with an order of exercise test in place of gait mode. The level of statistical significance was set at *p* = 0.05. The SPSS (Chicago, IL, USA, v. 25) was used for all analyses.

## 3. Results

### 3.1. Characteristics of Participants

Age, body mass, height, body mass index, body fat, and thickness of fat layers over the VL and GM muscles are presented in Table 1.

### 3.2. Heart Rate

**Figure 1:**
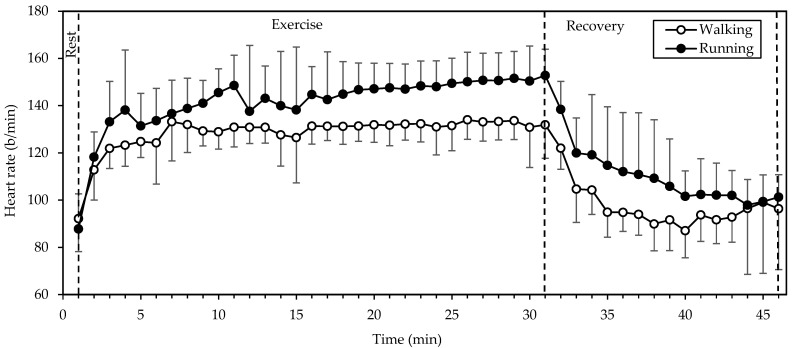
Mean and standard deviation of heart rate, averaged every minute, at rest, exercise, and recovery in walking and running. There were significant main effects of gait mode and time (both *p* < 0.001).

HR (Figure 1) exhibited significant main effects of gait mode and time (both *p* < 0.001; η^2^ = 0.769 and 0.803, respectively). Values were higher in running compared to walking throughout the exercise and recovery periods and exhibited a rapid increase from rest to the 3rd minute of exercise. Thereafter they remained relatively stable (with a slight incremental trend) until the end of exercise and decreased exponentially during recovery. Walking was performed at 68 ± 5% of the predicted maximal HR (HRmax), that is, 207 − 0.7 × age [13], while running was performed at 75 ± 7% of HRmax. HR during running was higher by 12 ± 10%, as compared to walking.

### 3.3. Respiratory Parameters and Energy Expenditure

**Figure 2:**
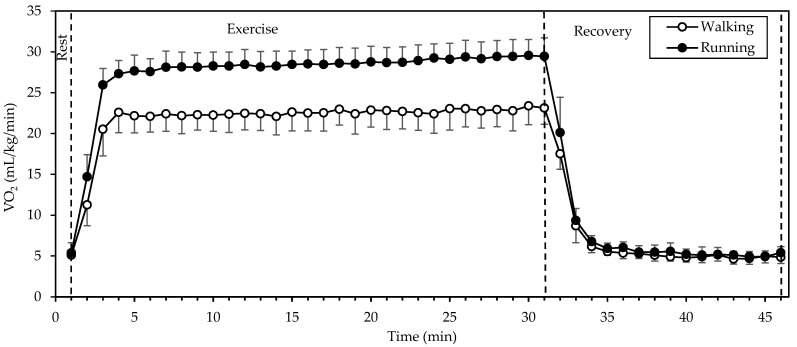
Mean and standard deviation of oxygen uptake, averaged every minute, at rest, exercise, and recovery in walking and running. There was a significant interaction of gait mode and time, as well as significant main effects of gait mode and time (all *p* < 0.001).

$\dot{V}O_{2}$ (Figure 2) showed a significant interaction of gait mode and time, as well as significant main effects of gait mode and time (all *p* < 0.001; η^2^ = 0.917, 0.992, and 0.740, respectively). Values were higher in running compared to walking during exercise, whereas they were identical or very similar between gait modes at rest and recovery. $\dot{V}O_{2}$ exhibited a sharp increase from rest to the 2nd minute of exercise and remained relatively stable until the end of exercise, decreasing sharply within 2 min of recovery. Walking during the period of stable $\dot{V}O_{2}$ (that is from the 3rd to the 30th minute of exercise) was performed at 22.6 ± 2.1 mL/kg/min, while running was performed at 28.6 ± 1.8 mL/kg/min. $\dot{V}O_{2}$ during running was higher by 27 ± 10%, as compared to walking.

Average RER during the period of stable $\dot{V}O_{2}$ (that is from the 3rd to the 30th minute of exercise) did not differ significantly between gait modes (*p* = 0.783) and was 0.881 ± 0.028. Average energy expenditure during the same period was 6.9 ± 0.7 kcal/kg/h in walking and 8.7 ± 0.5 kcal/kg/h in running (*p* < 0.001). Energy expenditure during running was higher by 27 ± 11%, as compared to walking.

### 3.4. Muscle Oxygenation

**Figure 3:**
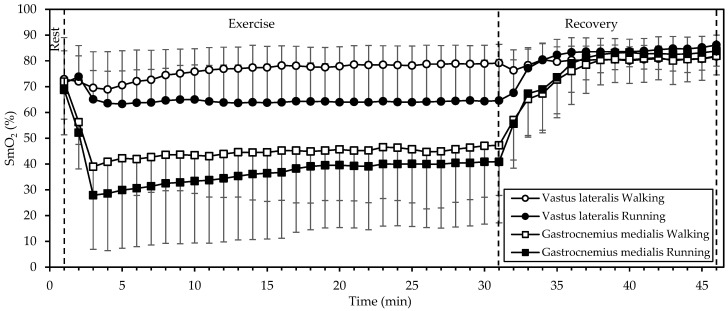
Mean and standard deviation of muscle oxygen saturation, averaged every minute, at rest, exercise, and recovery in walking and running. There was a significant interaction of gait mode, muscle, and time, as well as significant main effects of muscle and time (all *p* < 0.01).

SmO_2_ (Figure 3) displayed significant interactions of gait mode, muscle, and time; gait mode and time; and muscle and time (all *p* < 0.001; η^2^ = 0.382, 0.349, and 0.732, respectively). There were also significant main effects of muscle (*p* = 0.004, η^2^ = 0.630) and time (*p* < 0.001, η^2^ = 0.901). Starting values in both muscles and in both gait modes did not differ significantly (*p* > 0.4); in fact, they were quite similar (averaging 71%). During exercise, SmO_2_ remained relatively stable in the VL. In the GM, SmO_2_ dropped sharply during the initial 3 min and increased slowly thereafter, while remaining below baseline. In addition, SmO_2_ was lower during running, compared to walking (*p* = 0.045, η^2^ = 0.374), averaging 64% vs. 76% in the VL and 37% vs. 45% in the GM. During recovery, SmO_2_ increased in both muscles and in both gait modes, reaching similar values at 15 min of recovery (averaging 83%, that is, well above baseline).

### 3.5. Muscle Blood Supply

**Figure 4:**
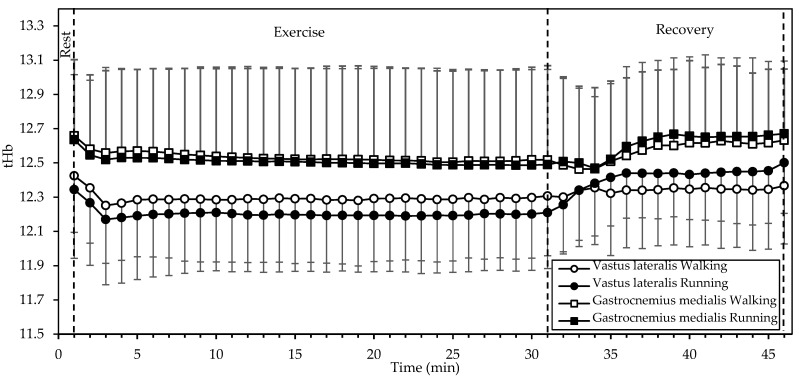
Mean and standard deviation of total hemoglobin, averaged every minute, at rest, exercise, and recovery in walking and running. There was a significant interaction of gait mode and time, as well as a significant main effect of time in both muscles (all *p* < 0.001).

tHb (Figure 4) showed significant interactions of gait mode and time, as well as a significant main effect of time in both the VL (both *p* < 0.001, η^2^ = 0.540 and 0.584, respectively) and the GM (both *p* < 0.001, η^2^ = 0.190 and 0.215, respectively). Values in both muscles decreased during the first two minutes of exercise and remained relatively constant until the end of exercise. During recovery, tHb increased, with a reversal between gait modes (that is, higher in running during recovery, as opposed to higher in walking during exercise).

### 3.6. Blood Lactate

**Figure 5:**
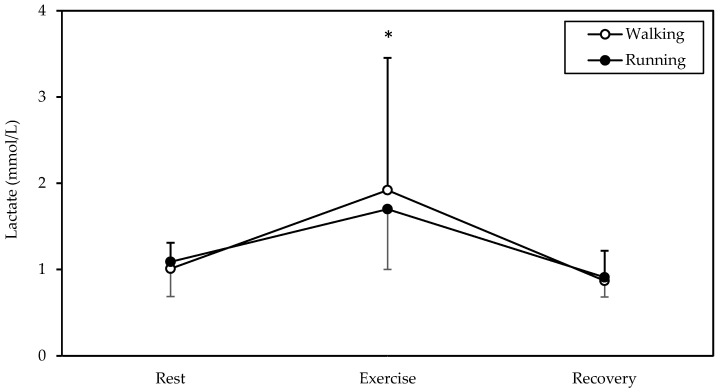
Mean and standard deviation of lactate at rest, exercise, and recovery in walking and running. * Significantly different from rest and recovery (*p* < 0.05).

A significant main effect of time was found in blood lactate (*p* = 0.009, η^2^ = 0.533, Figure 5). Lactate was higher in exercise (averaging 1.8 mmol/L) compared to rest and recovery (both *p* < 0.05).

### 3.7. Order Effect

There was no significant order effect on the outcome measures of this study, except for RER and tHb in the VL (both *p* < 0.001). RER was higher in the first gait mode (0.90 ± 0.03) compared to the second one (0.86 ± 0.03). tHb was lower in the first gait mode (12.03 ± 0.42) compared to the second one (12.13 ± 0.40).

## 4. Discussion

This study examined, for the first time, differences in the muscle oxygenation and blood supply of two leg muscles (VL and GM) between two gait modes (walking and running) at the same speed—one that was comfortable for both gait modes. Compared to whole-body oxygen uptake kinetics, the study of local oxygen utilization (that is, at the level of the exercising muscles via changes in SmO_2_) during exercise offers the advantage of a more focused and detailed examination of exercise metabolism, discerning between different muscles, and exploring the effects of manipulations such as blood flow restriction. Our main finding is that running caused higher muscle deoxygenation (accompanied by higher whole-body oxygen uptake, energy expenditure, and heart rate) than walking, all with large effect sizes, thus pointing to a higher internal load despite equal external load. This finding suggests that opting for running instead of walking at the same speed could lead to increased local muscle deoxygenation, which may be beneficial in inducing favorable adaptations to training.

Regarding the exercise parameters, we chose a typical duration of endurance exercise (30 min) and an intensity (7 km/h) that allowed the participants to perform both walking and running comfortably. The absence of an order effect in HR, $\dot{V}O_{2}$, SmO_2_, and blood lactate shows that the 15 min interval between the two tests was adequate for the participants to recover and be ready for the next test. The coincidence of baseline values in Figure 1, Figure 2, Figure 3 and Figure 5 testifies to this. No warm-up was included in the study protocol, as the exercise intensity was moderate.

Our finding of a higher energy expenditure of running vs. walking at 7 km/h agrees with that of Margaria et al. [14] In fact, the energy expenditure of walking and running at 7 km/h on a horizontal treadmill resulting from their Figure 1 (6.3 and 8.0 kcal/kg/h, respectively) is similar to our data (6.9 and 8.7 kcal/kg/h, see Results above). Thus, preferring running over walking at the same speed may be useful in weight management, as increased energy expenditure is the primary way in which exercise fights obesity [15]. In line with this, a prospective study with 47,453 participants demonstrated greater weight loss from running than walking the same distance daily [16]. As for the energy sources used, there seems to have been no difference between gait modes, as judged from the lack of significant differences in RER or blood lactate. The average RER value of 0.881 points to an aerobic energy contribution of 62% from carbohydrates and 38% from lipids [11].

The higher energy cost of running may be explained by the greater vertical displacement of the center of body mass with each stride, which necessitates a more forceful concentric and eccentric activity of leg muscles. Additionally, biomechanical differences [8,17], and differences in the timing of muscle activation between gait types [9] may play a role. As for the higher HR values during running, they may be explained by a higher stimulation of foot mechanoreceptors that provide feedback to cardiovascular control areas in the brain stem [18].

Although it is known that running requires a higher whole-body oxygen consumption than walking at speeds that are comfortable for both gait modes, such information is lacking at the level of muscle. Thus, our finding of a higher deoxygenation of two leg muscles during running is novel and shows that the higher $\dot{V}O_{2}$ during running was not able to compensate for the higher oxygen demand in the muscles, although exercise was of moderate intensity. What is more, this finding locates the difference (at least in part) at the exercising muscles and corroborates the hypothesis (described above) of higher muscle activity as an explanation for the higher energy cost of running. Additionally, the higher muscle deoxygenation during running vs. walking shows that the difference in energy demand between the two gait modes at the same speed is higher than the one thought so far on the basis of the difference in oxygen uptake.

The local muscle deoxygenation seen in this study, mainly during running, may have implications for inducing favorable adaptations mediated by hypoxia-inducible factor 1 (HIF-1). It is known that HIF-1 is a transcription factor which improves tissue function during low oxygen availability by inducing genes involved in oxygen transport, glucose transport, and glycolysis [19]. Thus, our study suggests that preferring running over walking at the same speed may result in adaptations favoring energy production.

While SmO_2_ was lower during running than walking in both muscles that we examined, there was a marked difference in that SmO_2_ remained relatively stable in the VL but dropped in the GM during exercise. Since blood supply (as estimated through tHb) to each muscle remained relatively constant throughout exercise (apart from a decrease during the first two minutes), one may conclude that the blood was generally able to match the increased oxygen demand of exercise in the VL but not in the GM. This difference may be due to the different activation or fiber composition of the two muscles. Johnson et al., [20] in an autopsy study, found that the GM had more type I fibers (50.8%) compared to the VL (42.3%; mean of surface and deep). Similarly, Edgerton et al. [21] found that the GM and VL contained about 50% and 32% slow twitch fibers, respectively. It is known that type I fibers are more oxidative, contain more mitochondria, and have a higher blood supply (capillary-to-fiber ratio) than type II fibers [22]. Hence, the drop in GM SmO_2_ during exercise may be explained by a higher use of oxygen to support aerobic energy production in the more abundant type I fibers, as compared to the VL.

Interestingly, SmO_2_ was rapidly restored (within up to 3 min) after exercise in all cases, exceeding the baseline afterwards (Figure 3). This response may be explained by the decrease in oxygen demand and the increase in blood supply (Figure 4) with the cessation of exercise.

Our findings regarding SmO_2_ (Figure 3) agree with those of Hiroyuki et al. [5]: Although they did not compare walking and running at the same speed, they found that, in walking (4 and 6 km/h), the oxygenation of the VL increased, whereas that of gastrocnemius lateralis decreased; then, in running (8 to 16 km/h), oxygenation decreased in both muscles. Similarly, Rissanen et al. [4] showed a linear decrease in VL oxygenation from walking (5 km/h) to running to exhaustion (15 km/h).

Our findings are delimited by the inclusion of male, healthy, fit, and normal-weight participants. Hence, these findings cannot be generalized to other populations, such as females, patients with chronic diseases, unfit individuals, and overweight or obese persons.

## 5. Conclusions

In young, healthy males, running caused higher VL and GM deoxygenation (along with higher whole-body oxygen uptake, energy expenditure, and heart rate) than walking at 7 km/h. This novel finding shows that the difference in energy demand between the two gait modes at the same speed is higher than the one documented in the literature based on the difference in $\dot{V}O_{2}$ alone. Future studies may examine whether these findings hold in women, in other ages, in disease states, and in designs with (experimentally induced or pathological) blood flow restriction, thus forming the foundation for health applications based on different gait modes.

Our study has the following practical implications:

- Preferring running over walking at the same speed results in higher energy expenditure, which may be useful in weight loss. It would be useful to inform interested parties that, in addition to the external load, the type of exercise may be a factor of choice in designing a weight management program.
- Preferring running over walking at the same speed causes higher local muscle deoxygenation, which may be beneficial in inducing favorable training adaptations of the leg muscles.
- Examining the oxygenation of different muscles during exercise may be useful in discerning differences in muscle activation and/or fiber type composition.

## Figures and Tables

**Table 1:** Characteristics of participants (mean ± SD, *n* = 10).

Age (years)	22.7 ± 1.6
Body mass (kg)	79.6 ± 7.5
Body height (m)	1.81 ± 0.05
Body mass index (kg/m^2^)	24.3 ± 1.7
Body fat (%)	14.3 ± 4.2
Fat layer over vastus lateralis (mm)	6.7 ± 1.7
Fat layer over gastrocnemius medialis (mm)	5.0 ± 1.8

## Data Availability

The data presented in this study are available on request from the corresponding author due to restrictions (ethical reasons).
